# Simplified molecular classification of lung adenocarcinomas based on *EGFR, KRAS,* and *TP53* mutations

**DOI:** 10.1186/s12885-020-6579-z

**Published:** 2020-01-31

**Authors:** Roberto Ruiz-Cordero, Junsheng Ma, Abha Khanna, Genevieve Lyons, Waree Rinsurongkawong, Roland Bassett, Ming Guo, Mark J. Routbort, Jianjun Zhang, Ferdinandos Skoulidis, John Heymach, Emily B. Roarty, Zhenya Tang, L. Jeffrey Medeiros, Keyur P. Patel, Rajyalakshmi Luthra, Sinchita Roy-Chowdhuri

**Affiliations:** 1 Department of Pathology, University of California San Francisco, 1825 4th Street, Room L2181A, San Francisco, CA 94158 USA; 2 Department of Thoracic/Head and Neck Medical Oncology, The University of Texas MD Anderson Cancer Center, Houston, TX USA; 3 Department of Hematopathology, The University of Texas MD Anderson Cancer Center, Houston, TX USA; 4 Department of Biostatistics, The University of Texas MD Anderson Cancer Center, Houston, TX USA; 5 Department of Pathology, The University of Texas MD Anderson Cancer Center, Houston, TX USA

**Keywords:** Lung adenocarcinoma, Next generation sequencing, Molecular subtypes

## Abstract

**Background:**

Gene expression profiling has consistently identified three molecular subtypes of lung adenocarcinoma that have prognostic implications. To facilitate stratification of patients with this disease into similar molecular subtypes, we developed and validated a simple, mutually exclusive classification.

**Methods:**

Mutational status of *EGFR*, *KRAS*, and *TP53* was used to define seven mutually exclusive molecular subtypes. A development cohort of 283 cytology specimens of lung adenocarcinoma was used to evaluate the associations between the proposed classification and clinicopathologic variables including demographic characteristics, smoking history, fluorescence in situ hybridization and molecular results. For validation and prognostic assessment, 63 of the 283 cytology specimens with available survival data were combined with a separate cohort of 428 surgical pathology specimens of lung adenocarcinoma.

**Results:**

The proposed classification yielded significant associations between these molecular subtypes and clinical and prognostic features. We found better overall survival in patients who underwent surgery and had tumors enriched for *EGFR* mutations. Worse overall survival was associated with older age, stage IV disease, and tumors with co-mutations in *KRAS* and *TP53*. Interestingly, neither chemotherapy nor radiation therapy showed benefit to overall survival.

**Conclusions:**

The mutational status of *EGFR*, *KRAS*, and *TP53* can be used to easily classify lung adenocarcinoma patients into seven subtypes that show a relationship with prognosis, especially in patients who underwent surgery, and these subtypes are similar to classifications based on more complex genomic methods reported previously.

## Background

Lung cancer is the main cause of cancer-related mortality in both men and women [1–3]. Lung adenocarcinoma accounts for approximately 40% of lung cancer cases [4–6]. Gene expression profiling of lung adenocarcinomas has consistently identified three molecular subtypes with prognostic implications [7–14]. The initial molecular classification of lung adenocarcinomas included the bronchoid, magnoid, and squamoid subtypes [12, 15]. However, after comprehensive molecular profiling of a cohort of lung adenocarcinomas, The Cancer Genome Atlas Research Network proposed an updated nomenclature for this molecular classification that encompasses previous histopathologic, anatomic, and mutational classifications [8]. This system re-designated the initial subtypes as the terminal respiratory unit, proximal-proliferative, and proximal-inflammatory subtypes, respectively [8].

Tumors with acinar, papillary, or lepidic histomorphology and mutations or copy number alterations in *EGFR*, presenting most often in women who have never smoked, predominantly cluster in the terminal respiratory unit subtype. Tumors in the proximal-proliferative subtype have variable histology and commonly display mutations and copy number alterations in *KRAS* and *STK11*. In contrast, lung adenocarcinomas with primarily solid architecture and enrichment for *TP53* and *NF1* mutations and *p16* methylation typically cluster in the proximal-inflammatory subtype [8, 15].

While molecular subtypes of lung adenocarcinoma have been associated with significant differences in prognosis, routine gene expression profiling in the clinical setting has been limited by cost, complexity, and increased turnaround time [16]. These limitations have led to the development of simplified prognostic models based on the expression of selected genes [9, 16]. However, many of these genes, such as *PTK7, CIT, SCNN1A, PTGES, ERO1A, ZWINT, DUSP6, MMD, STAT1, ERBB3,* and *LCK*, are not tested routinely in the clinical laboratory.

To fill this need**,** we developed a simplified molecular subtype classification based on the mutational status of only *EGFR, KRAS*, and *TP53* to facilitate categorization of patients’ lung adenocarcinomas into molecular subtypes with relevant prognostic information.

## Materials and methods

### Patient selection for development cohort

We retrospectively reviewed our institutional database for patients treated between May 1, 2010, and October 31, 2015, to identify cytologic specimens of patients with lung adenocarcinoma. Patients with TTF1-negative non–small cell lung cancer, small cell lung cancer, large cell carcinoma, squamous carcinoma, and poorly differentiated carcinoma not otherwise specified were excluded. We reviewed the patients’ medical records for demographic characteristics, clinical information, fluorescence in situ hybridization (FISH) results for *ALK*, *ROS1*, *MET*, and/or *RET*, and mutation profiling data derived by next-generation sequencing (NGS) and polymerase chain reaction (PCR)-based methods (i.e. Sanger sequencing or pyrosequencing). PCR-based methods were restricted to analysis of only *EGFR*, *KRAS*, and *BRAF* hotspots (Supplement Data).

### Patient selection for validation cohort

Patients from our institution’s Genomic Marker-Guided Therapy Initiative (GEMINI) project database were selected as a validation cohort. This group included patients who underwent computerized tomography–guided transthoracic core-needle biopsy for diagnosis and/or staging of lung adenocarcinomas as well as patients who underwent surgery to resect lung adenocarcinoma between November 1, 2009, and October 31, 2016. Age, sex, race/ethnicity, smoking status, NGS mutation data, survival status, and treatment information were included in the analysis. To avoid Simpson’s paradox [17], we combined this cohort with a subset of cytology cases from the development cohort whose medical record numbers matched to those of records in the GEMINI database and who had available survival information and treatment data.

### Mutational analysis

NGS was performed on cytology smears or formalin-fixed paraffin-embedded tissue (cytology cell blocks or core biopsy tissue blocks) using the Ion Torrent or Ion Proton (Thermo Fisher Scientific) sequencers in our College of American Pathologists–accredited, Clinical Laboratory Improvement Amendments–certified laboratory. Multiple NGS panels were developed, validated, and implemented in our laboratory during the study period (2009–2016), including an initial hotspot panel of 46 cancer-related genes [18], an updated 50-gene hotspot panel, a 126-gene panel, and a panel of 409 cancer-associated genes [19]. The cytology specimens were appropriately validated [20]. All these panels include several amplicons targeting known hotspots in exons of *EGFR*, *KRAS*, and *TP53*.

### Simplified classification of molecular subtypes

We stratified cases from our development and validation cohorts by creating a classification system using the mutational status of *EGFR*, *KRAS*, and *TP53*, forming mutually exclusive groups. Cases that harbored mutations in *EGFR* only or mutations in *EGFR* and genes other than *KRAS* and *TP53* were classified as the simplified terminal respiratory unit (sTRU) subtype. Cases with *KRAS* mutations only or mutations in *KRAS* and genes other than *EGFR* and *TP53* were classified as the simplified proximal-proliferative (sPP) subtype. Cases with only *TP53* mutations or mutations in *TP53* and genes other than *EGFR* and *KRAS* were classified as the simplified proximal-inflammatory (sPI) subtype. Also, cases with co-mutations in *KRAS* and *TP53* (*KRAS/TP53* subtype) or *EGFR* and *TP53* (*EGFR/TP53* subtype) were grouped separately. Cases with mutations in genes other than *EGFR*, *KRAS*, and *TP53* were classified as the non-TRUPPPI subtype, and a few cases that lacked mutations in any of the genes detected by our NGS panels were placed in a “no-mutation” subtype.

## Statistical methods

### Development cohort

Categorical variables were summarized by frequencies and percentages, and continuous variables were summarized using means, standard deviations, medians, and ranges. Fisher exact test or its generalization for categorical variables was used to compare categorical variables between one molecular subtype and the remaining patients; in addition, Monte Carlo simulation approach was used when computational issues were encountered. Patients with indeterminate FISH results or unknown aneuploidy status were excluded from the Fisher exact tests. The Wilcoxon rank sum test or Kruskal-Wallis rank-sum test was used to compare continuous variables between molecular subtypes.

### Validation cohort

Associations between variables and subtypes were assessed as described for the development cohort. The outcome variable of overall survival (OS) time was computed from the date of initial diagnosis to the last follow-up date or death date. For the subset of patients who had surgery, separate analyses were performed of OS from the date of surgery. Cox proportional hazards models were used to evaluate associations of variables with survival outcomes, and Firth penalized Cox regression models were fitted for covariates with zero count of events. In multivariable Cox regression analyses, we included covariates that had *p* values less than 0.25 in univariate Cox regression models. Treatment variables (surgery, radiation, and chemotherapy) were handled as time-varying covariates. The Kaplan-Meier method was used to estimate survival distributions, and the log-rank test was used for comparisons between survival distributions. All statistical analyses were performed using R version 3.3.11 [21] and SAS version 9.4. All statistical tests used a significance level of 5%, and no adjustments for multiple testing were made.

## Results

### Development cohort

We collected a development cohort of 283 consecutive cytology samples from patients with lung adenocarcinoma. The samples were acquired via endobronchial ultrasound–guided FNA (64.7%, *n* = 183), thoracentesis and paracentesis (16.6%, *n* = 47 [46 pleural samples]), computed tomography–guided FNA (13.4%, *n* = 38), and ultrasound-guided FNA (5.3%, *n* = 15); most samples were cell block preparations (97.8%, *n* = 277). Metastases accounted for 82.7% (*n* = 234) of cases, and the majority were to lymph nodes (66.7%, *n* = 156), followed by pleural fluid (20.1%, *n* = 47), soft tissue (3.8%, *n* = 9), bones (3.4%, *n* = 8), adrenal glands (3.0%, *n* = 7 [5 on the left]), liver (2.1%, *n* = 5), and other sites (0.9%, *n* = 2). The relevant demographic characteristics and clinicopathologic data for the development cohort are summarized in Table 1. The cohort was composed primarily of older individuals with a median age of 65.4 years (range: 27.5–90.2 years) and 151 (53.4%) women. Most patients were white, current or former smokers, and had stage IV disease at the time of data collection. All cases underwent FISH testing for *ALK*, *ROS1*, *MET*, and/or *RET.* The FISH results were negative in 250 (88.3%) cases. The rest of the cases were positive for rearrangements or amplification of *ALK* (5.7%, *n* = 16), *MET* (1.8%, *n* = 5), *RET* (0.7%, *n* = 2), or *ROS1* (0.3%, n = 1), or were indeterminate (3.2%, *n* = 9). Aneuploidy, defined as an increase or decrease in the number of fluorescent signals observed in a cell, was present in 193 (68.2%) cases, not present in 60 (21.2%), indeterminate in 27 (9.5%), and not assessed in three (1.1%) cases.

**Table 1:** Demographic and clinicopathologic characteristics in the development cohort

Variable	Value
Sex, n (%)	
Male	132 (46.6)
Female	151 (53.4)
Age, median (range)	65.4 y (27.5–90.2 y)
Race/ethnicity, n (%)	
Caucasian	215 (76.0)
Asian	27 (9.5)
Black	19 (6.7)
Hispanic	21 (7.4)
Unknown	1 (0.3)
Smoking status, n (%)	
Never-smoker	64 (22.6)
Former smoker	136 (48.1)
Current smoker	82 (29.0)
Unknown	1 (0.3)
Vital status, n (%)	
Alive	172 (60.8)
Deceased	111 (39.2)
Molecular platform, n (%)	
NGS	218 (77.0)
PCR^a^	55 (19.5)
Not done	10 (3.5)
Molecular subtype, n (%) (*n* = 233)	
sTRU – *EGFR* (%)	34 (14.6)
sPP – *KRAS* (%)	43 (18.5)
sPI – *TP53* (%)	46 (19.7)
Co-mutation	60 (25.8)
*EGFR/TP53*	26 (11.2)
*KRAS/ TP53*	34 (14.6)
Non-TRUPPPI	21 (9.0)
No mutations detected by NGS	29 (12.4)
FISH results, n (%)	
Negative	250 (88.3)
Positive	24 (8.5)
*ALK*	16 (5.7)
*ROS1*	1 (0.4)
*RET*	2 (0.7)
*MET*	5 (1.8)
Indeterminate	9 (3.2)
Aneuploidy	193 (68.2)

*NGS* Next-generation sequencing, *PCR* Polymerase chain reaction, *sTRU* Simplified terminal respiratory unit, *sPP* Simplified proximal-proliferative, *sPI* Simplified proximal-inflammatory, *non-TRUPPPI* Mutations in genes other than *EGFR*, *KRAS*, and *TP53*, *FISH* Fluorescence in situ hybridization

^a^Sanger sequencing or pyrosequencing

Mutational analysis, including NGS and PCR-based methods, was performed in 273 (96.5%) cases. NGS was performed in 77% (*n* = 218) of the specimens, yielding positive mutations in 188 (86.2%) cases. PCR-based testing was performed in 55 (20%) cases, yielding positive mutations in 15 (27.3%) (9 in *EGFR*, 6 in *KRAS*, and 0 in *BRAF*). Of the cases with PCR-based testing, two cases had inadequate DNA material, and 38 cases were negative for single-gene testing. These 40 cases as well as 10 cases in which mutational analysis was not performed were excluded from further analysis, leaving 233 cases. Mutations were most frequent in *TP53* (46.0%, *n* = 107), *KRAS* (33.5%, *n* = 78), and *EGFR* (26.2%, *n* = 61). According to our proposed classification, 34 (14.6%) cases were classified as sTRU, 43 (18.5%) as sPP, and 46 (19.7%) as sPI. Cases with co-mutations included 26 (11.2%) with *EGFR/TP53* and 34 (14.6%) with *KRAS/TP53*. There were 21 (9.0%) cases with mutations in genes other than *EGFR*, *KRAS*, and *TP53* (non-TRUPPPI subtype) and 29 (12.4%) cases with no mutations detected.

The simplified molecular subtypes were statistically significantly associated with age, race/ethnicity, smoking status, and aneuploidy. Table 2 summarizes the associations between molecular subtypes and clinicopathologic variables in the overall development cohort and each particular subtype. To identify further associations, we compared variables between patients within a given molecular subtype and the remaining patients (e.g. sTRU vs rest). The sTRU subtype was associated with Asian race/ethnicity (23.5% vs. 7.6%, *p* = 0.027) and never-smoker status (52.9% vs. 18.7%, *p* < 0.001). The sPP subtype was associated with white race/ethnicity (86.0% vs. 73.0%, *p* = 0.042). The sPI subtype was associated with male sex (63.0% vs. 41.2%, *p* = 0.008). The *EGFR/TP53* subtype was associated with younger age (mean age 56.9 vs. 65.8 years, *p* < 0.001), Asian (19.2% vs. 8.7%) and Hispanic race/ethnicity (19.2% vs. 6.3%, *p* = 0.026), never-smoker status (46.2% vs. 20.9%, *p* = 0.016), and lack of aneuploidy (60.0% vs. 80.1%, *p* = 0.038). The *KRAS/TP53* subtype was associated with current smoking (55.9% vs. 21.7%, *p* < 0.001). The non-TRUPPPI subtype was not associated with any of the covariates, and the no-mutation subgroup was associated with never-smoker status (41.4% vs. 21.2%, *p* = 0.045) and aneuploidy (95.8% vs. 75.3%, *p* = 0.019).

**Table 2:** Associations between molecular subtypes and clinicopathologic variables in the development cohort

Variable	N	Overall	sTRU (*n* = 34)	sPP (*n* = 43)	sPI (*n* = 46)	*EGFR/TP53* (*n* = 26)	*KRAS/TP53* (*n* = 34)	Non-TRUPPPI (*n* = 21)	No mutation (*n* = 29)	*P*
Age, mean (standard deviation), y	233	64.8 (10.9)	67.3 (10.6)	67.2 (9.0)	65.3 (9.3)	56.9 (12.6)	64.3 (7.9)	67.5 (12.5)	62.9 (13.6)	0.005
Sex, n (%)	233									0.075
Female		127 (54.5)	20 (58.8)	27 (62.8)	17 (37.0)	17 (65.4)	20 (58.8)	8 (38.1)	18 (62.1)	
Male		106 (45.5)	14 (41.2)	16 (37.2)	29 (63.0)	9 (34.6)	14 (41.2)	13 (61.9)	11 (37.9)	
FISH, n (%)	226									0.544
Negative		206 (91.2)	27 (81.8)	37 (88.1)	43 (93.5)	23 (92.0)	30 (93.8)	20 (95.2)	26 (96.3)	
Positive		20 (8.8)	6 (18.2)	5 (11.9)	3 (6.5)	2 (8.0)	2 (6.2)	1 (4.8)	1 (3.7)	
Race/ethnicity, n (%)	232									0.041^a^
Asian		23 (9.9)	8 (23.5)	0 (0.0)	5 (10.9)	5 (19.2)	1 (3.0)	2 (9.5)	2 (6.9)	
Black		16 (6.9)	3 (8.8)	2 (4.7)	5 (10.9)	1 (3.8)	2 (6.1)	2 (9.5)	1 (3.4)	
White		175 (75.4)	20 (58.8)	37 (86.0)	33 (71.7)	15 (57.7)	29 (87.9)	16 (76.2)	25 (86.2)	
Hispanic		18 (7.8)	3 (8.8)	4 (9.3)	3 (6.5)	5 (19.2)	1 (3.0)	1 (4.8)	1 (3.4)	
Smoking, n (%)	232									*<* 0.001^a^
Current		62 (26.7)	5 (14.7)	12 (27.9)	14 (31.1)	3 (11.5)	19 (55.9)	5 (23.8)	4 (13.8)	
Former		115 (49.6)	11 (32.4)	26 (60.5)	25 (55.6)	11 (42.3)	15 (44.1)	14 (66.7)	13 (44.8)	
Never		55 (23.7)	18 (52.9)	5 (11.6)	6 (13.3)	12 (46.2)	0 (0.0)	2 (9.5)	12 (41.4)	
Aneuploidy, n (%)	206									0.025
No		46 (22.3)	11 (35.5)	9 (23.1)	9 (20.9)	10 (40.0)	3 (12.0)	3 (15.8)	1 (4.2)	
Yes		160 (77.7)	20 (64.5)	30 (76.9)	34 (79.1)	15 (60.0)	22 (88.0)	16 (84.2)	23 (95.8)	

*sTRU* Simplified terminal respiratory unit, *sPP* Simplified proximal-proliferative, *sPI* Simplified proximal-inflammatory, non-TRUPPPI Mutated in genes other than *EGFR*, *KRAS*, and *TP53*, *FISH* Fluorescence in situ hybridization

*P* values are based on Fisher exact test for categorical variables and Kruskal-Wallis rank-sum test for continuous variables

^a^Fisher exact test with Monte Carlo simulation

### Validation cohort

To validate these findings and determine the impact of our subtypes on prognosis, we used a validation cohort (*n* = 428) composed of core-needle biopsy samples or resection specimens from lung adenocarcinoma patients with available data on treatment and follow-up. Histomorphologic subtypes (e.g., mucinous, lepidic, acinar, and solid) were reported in 28.3% (*n* = 121) of the pathology reports. The mutational data for this cohort were based only on NGS because all three target genes were not assessed in cases where PCR-based single-gene analysis was performed. Also, we included the 63 patients from the cytology cohort in the GEMINI database with treatment and follow-up data available. NGS results were available for 85.7% (*n* = 54) of these cases.

### Mutational profiling of lung adenocarcinoma patients in the validation cohort

**Fig. 1:**
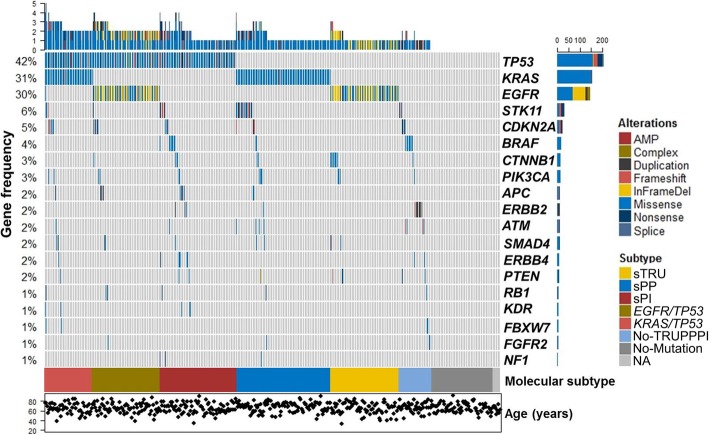
Oncoprint plot of the validation cohort. The mutational profile of the 491 patients in the validation cohort including the 19 most commonly mutated genes is shown. Each column represents a patient. The upper histogram highlights the number of genes mutated in each case. Mutated genes are in descending order of frequency, and their mutation frequency is shown on the y axis. Below the columns, the color map indicates the simplified molecular subtypes. At the bottom, the dot plot shows the age of each patient. The horizontal bars adjacent to the genes illustrate the number and type of genetic alterations. AMP, amplification; InFrameDel, in-frame deletion; NA, sequencing not available

Sequencing data were available for 484 (98.6%) patients in the combined validation cohort (Supplement Data). NGS and PCR analyses yielded a total of 835 mutations/variants in 421 patients (87.0%). The median tumor percentage was 40% (range: 20 to 95% tumor cells). Most of the genomic alterations were missense mutations (75%, *n* = 618), followed by in-frame deletions (7.0%, *n* = 58), nonsense (6.6%, *n* = 55) and frameshift (5.0%, *n* = 40) mutations, duplications (2.1%, *n* = 18), complex mutations/indels (1.8%, *n* = 15), splice mutations (1.4%, *n* = 12), and gene amplifications (1.1%, *n* = 10). Transversions included G > T (27%, *n* = 222), T > G (7.0%, *n* = 60), C > A (1.5%, *n* = 13), and A > C (1.0%, *n* = 8), and transitions included G > A (13%, *n* = 106), C > T (12%, *n* = 99), A > G (4.0%, *n* = 34), and T > C (1.0%, n = 9). The most common protein alterations were *KRAS*-G12C (*n* = 58), *EGFR*-L858R (*n* = 51), *EGFR*-E746_A750del (*n* = 45), *KRAS*-G12 V (*n* = 36), *KRAS*-G12D (*n* = 27), and *EGFR*-T790 M (n = 27). The mutational data for all 491 cases in the validation cohort, stratified by simplified molecular subtype, are summarized in Fig. 1.

### Clinical and histomorphologic associations according to simplified molecular subtypes

The simplified molecular subtypes were significantly associated with age, race/ethnicity, sex, smoking status, stage, histomorphology and FISH results. Table 3 summarizes the associations between molecular subtypes and clinicopathologic variables in the overall validation cohort and each particular subtype. As in the development cohort, variables were compared between patients within a given molecular subtype and the remaining patients. The sTRU subtype was associated with slightly older age (mean age 66.4 vs. 63.2 years, *p* = 0.015), never-smoker status (62.2% vs. 26.3%, *p* < 0.001) Asian race/ethnicity (17.6% vs. 6.6%, *p* = 0.022), metastatic tumors (61.5% vs. 41.7%, *p* = 0.013), non-mucinous (95.5% vs. 70.7%, *p* = 0.014) and lepidic histology (63.6% vs. 36.4%, *p* = 0.030), and stage IV disease (55.4% vs. 41.6%, *p* = 0.007). The sPP subtype was associated with slightly older age (65.9 vs. 63.1 years, *p* = 0.026), lower likelihood of never-smoker status (10.8% vs. 37.4%, *p* < 0.001), black race/ethnicity (10.8% vs 6.5%, *p* = 0.033), tumors with mucinous (42.9% vs. 17.4%, *p* = 0.005) and non-acinar (80.0% vs. 58.1%, *p* = 0.035) histology, non-metastatic tumors (69.1% vs 50.9%, *p* = 0.016), negativity for *ALK/ROS1/MET/RET* abnormalities (96.8% vs 86.6%, *p* = 0.003), and stage II disease (18.6% vs. 7.1%, *p* < 0.001). The sPI molecular subtype was associated with male sex (55.4% vs. 39.4%, *p* = 0.01), lower likelihood of never-smoker status (16.9% vs. 34.9%, *p* = 0.003), and stage III disease (32.5% vs. 20.2%, *p* = 0.047). Like the sTRU subtype, the *EGFR*/*TP53* subtype was associated with younger age (mean age 59.1 vs. 64.5 years, *p* < 0.001), Asian race/ethnicity (18.9% vs. 6.3%, *p* < 0.001), never-smoker status (55.4% vs. 27.6%, *p* < 0.001), and smaller tumors (mean tumor size 3.27 cm vs. 3.96 cm, *p* = 0.042). In contrast, the *KRAS*/*TP53* subtype was associated with Hispanic race/ethnicity (12.0% vs. 5.8%, *p* = 0.035), lower likelihood of never-smoker status (6.0% vs. 34.8%, *p* < 0.001), and solid (45.5% vs. 11.8%, *p* = 0.011) and non-lepidic (90.9% vs. 55.5%, *p* = 0.026) histology. The non-TRUPPPI subtype was associated with mucinous histology (62.5% vs. 22.1%, *p* = 0.022), whereas the no-mutation subtype was associated with never-smoker status (46.0% vs. 29.7%, *p* = 0.035) and acinar histology (57.1% vs. 31.0%, *p* = 0.043). No significant associations were identified between subtype and alcohol intake.

**Table 3:** Associations between molecular subtypes and clinicopathologic variables in the validation cohort

Variable	*N*	Overall	sTRU (*n* = 74)	sPP (*n* = 102)	sPI (*n* = 83)	*EGFR/TP53* (*n* = 74)	*KRAS/TP53* (*n* = 50)	Non-TRUPPPI (*n* = 38)	No mutation (*n* = 63)	*P*
Age, mean (standard deviation), y	484	63.7 (10.4)	66.4 (9.1)	65.9 (9.4)	63.2 (9.3)	59.1 (11.9)	64.1 (8.5)	63.7 (11.7)	62.7 (12.2)	*<* 0.001
Race/ethnicity, n (%)	484									*<* 0.001^a^
Asian		40 (8.3)	13 (17.6)	2 (2.0)	4 (4.8)	14 (18.9)	0 (0.0)	1 (2.6)	6 (9.5)	
Black		36 (7.4)	2 (2.7)	11 (10.8)	6 (7.2)	6 (8.1)	3 (6.0)	2 (5.3)	6 (9.5)	
White		372 (76.9)	54 (73.0)	80 (78.4)	71 (85.5)	44 (59.5)	40 (80.0)	35 (92.1)	48 (76.2)	
Hispanic		31 (6.4)	5 (6.8)	8 (7.8)	2 (2.4)	7 (9.5)	6 (12.0)	0 (0.0)	3 (4.8)	
Other		5 (1.0)	0 (0.0)	1 (1.0)	0 (0.0)	3 (4.1)	1 (2.0)	0 (0.0)	0 (0.0)	
Sex, n (%)	484									0.022
Female		280 (57.9)	49 (66.2)	65 (63.7)	37 (44.6)	43 (58.1)	32 (64.0)	25 (65.8)	29 (46.0)	
Male		204 (42.1)	25 (33.8)	37 (36.3)	46 (55.4)	31 (41.9)	18 (36.0)	13 (34.2)	34 (54.0)	
Smoking, n (%)	484									*<* 0.001^a^
Current		60 (12.4)	2 (2.7)	22 (21.6)	14 (16.9)	4 (5.4)	8 (16.0)	3 (7.9)	7 (11.1)	
Former		270 (55.8)	26 (35.1)	69 (67.6)	55 (66.3)	29 (39.2)	39 (78.0)	25 (65.8)	27 (42.9)	
Never		154 (31.8)	46 (62.2)	11 (10.8)	14 (16.9)	41 (55.4)	3 (6.0)	10 (26.3)	29 (46.0)	
Alcohol, n (%)	478									0.476^a^
Current		174 (36.4)	30 (41.1)	36 (35.3)	29 (36.2)	26 (35.1)	16 (32.0)	16 (42.1)	21 (34.4)	
Former		91 (19.0)	12 (16.4)	21 (20.6)	14 (17.5)	7 (9.5)	14 (28.0)	10 (26.3)	13 (21.3)	
Never		177 (37.0)	28 (38.4)	35 (34.3)	31 (38.8)	33 (44.6)	17 (34.0)	12 (31.6)	21 (34.4)	
Social		36 (7.5)	3 (4.1)	10 (9.8)	6 (7.5)	8 (10.8)	3 (6.0)	0 (0.0)	6 (9.8)	
Tumor size, mean (standard deviation), cm	449	3.86 (2.28)	3.78 (2.05)	4.36 (2.81)	3.80 (2.16)	3.27 (1.69)	3.97 (2.20)	3.43 (2.00)	4.05 (2.44)	0.234
Metastatic, n (%)	275									0.018
No		150 (54.5)	20 (38.5)	38 (69.1)	20 (47.6)	20 (45.5)	16 (59.3)	12 (66.7)	24 (64.9)	
Yes		125 (45.5)	32 (61.5)	17 (30.9)	22 (52.4)	24 (54.5)	11 (40.7)	6 (33.3)	13 (35.1)	
Mucinous, n (%)	121									< 0.001
0		91 (75.2)	21 (95.5)	20 (57.1)	11 (78.6)	10 (100.0)	11 (100.0)	3 (37.5)	15 (71.4)	
1		30 (24.8)	1 (4.5)	15 (42.9)	3 (21.4)	0 (0.0)	0 (0.0)	5 (62.5)	6 (28.6)	
Lepidic, n (%)	121									0.063
0		71 (58.7)	8 (36.4)	21 (60.0)	10 (71.4)	7 (70.0)	10 (90.9)	5 (62.5)	10 (47.6)	
1		50 (41.3)	14 (63.6)	14 (40.0)	4 (28.6)	3 (30.0)	1 (9.1)	3 (37.5)	11 (52.4)	
Acinar, n (%)	121									0.012
0		78 (64.5)	17 (77.3)	28 (80.0)	8 (57.1)	4 (40.0)	5 (45.5)	7 (87.5)	9 (42.9)	
1		43 (35.5)	5 (22.7)	7 (20.0)	6 (42.9)	6 (60.0)	6 (54.5)	1 (12.5)	12 (57.1)	
Micro/papillary, n (%)	121									0.175
0		106 (87.6)	21 (95.5)	31 (88.6)	14 (100.0)	7 (70.0)	10 (90.9)	6 (75.0)	17 (81.0)	
1		15 (12.4)	1 (4.5)	4 (11.4)	0 (0.0)	3 (30.0)	1 (9.1)	2 (25.0)	4 (19.0)	
Solid, n (%)	121									0.143
0		103 (85.1)	20 (90.9)	30 (85.7)	11 (78.6)	9 (90.0)	6 (54.5)	8 (100.0)	19 (90.5)	
1		18 (14.9)	2 (9.1)	5 (14.3)	3 (21.4)	1 (10.0)	5 (45.5)	0 (0.0)	2 (9.5)	
Mixed, n (%)	121									0.492
0		88 (72.7)	19 (86.4)	26 (74.3)	9 (64.3)	8 (80.0)	8 (72.7)	6 (75.0)	12 (57.1)	
1		33 (27.3)	3 (13.6)	9 (25.7)	5 (35.7)	2 (20.0)	3 (27.3)	2 (25.0)	9 (42.9)	
Primary, n (%)	428									0.056
0		233 (54.4)	39 (56.5)	40 (47.1)	41 (57.7)	30 (46.2)	21 (45.7)	22 (62.9)	40 (70.2)	
1		195 (45.6)	30 (43.5)	45 (52.9)	30 (42.3)	35 (53.8)	25 (54.3)	13 (37.1)	17 (29.8)	
Stage, n (%)	483									< 0.001^a^
I		118 (24.4)	20 (27.0)	26 (25.5)	14 (16.9)	19 (26.0)	16 (32.0)	11 (28.9)	12 (19.0)	
II		46 (9.5)	1 (1.4)	19 (18.6)	5 (6.0)	4 (5.5)	4 (8.0)	4 (10.5)	9 (14.3)	
III		108 (22.4)	12 (16.2)	29 (28.4)	27 (32.5)	11 (15.1)	9 (18.0)	5 (13.2)	15 (23.8)	
IV		211 (43.7)	41 (55.4)	28 (27.5)	37 (44.6)	39 (53.4)	21 (42.0)	18 (47.4)	27 (42.9)	
FISH, n (%)	453									0.042
Negative		402 (88.7)	64 (90.1)	92 (96.8)	65 (83.3)	63 (88.7)	35 (81.4)	32 (86.5)	51 (87.9)	
Positive		51 (11.3)	7 (9.9)	3 (3.2)	13 (16.7)	8 (11.3)	8 (18.6)	5 (13.5)	7 (12.1)	

*sTRU* Simplified terminal respiratory unit, *sPP* Simplified proximal-proliferative, *sPI* Simplified proximal-inflammatory, *non-TRUPPPI* Mutations in genes other than *EGFR*, *KRAS*, and *TP53*, *FISH* Fluorescence in situ hybridization

*P* values are based on Fisher exact test for categorical variables and Kruskal-Wallis rank-sum test for continuous variables

^a^Fisher exact test with Monte Carlo simulation

### Prognostic associations according to simplified molecular subtype classification

**Fig. 2:**
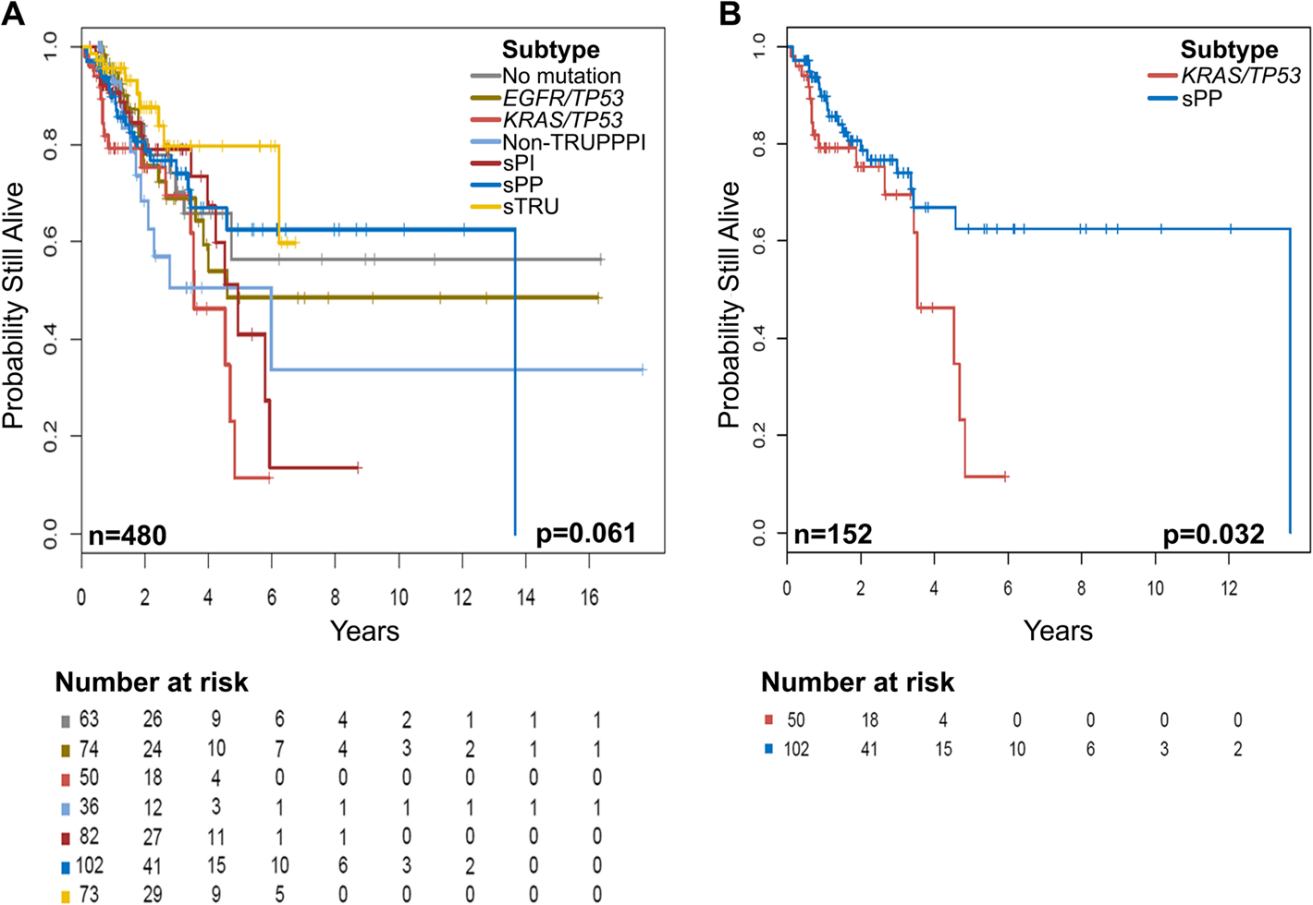
Overall survival (OS) of the entire validation cohort stratified by simplified molecular subtypes. Kaplan-Meier plots show the prognostic significance of the simplified molecular subtypes. **a** OS differs between the simplified molecular subtypes. **b** OS differs significantly between the sPP and *KRAS/TP53* subtypes. The number of patients and the log-rank test *p* value are shown at the bottom of each plot

**Fig. 3:**
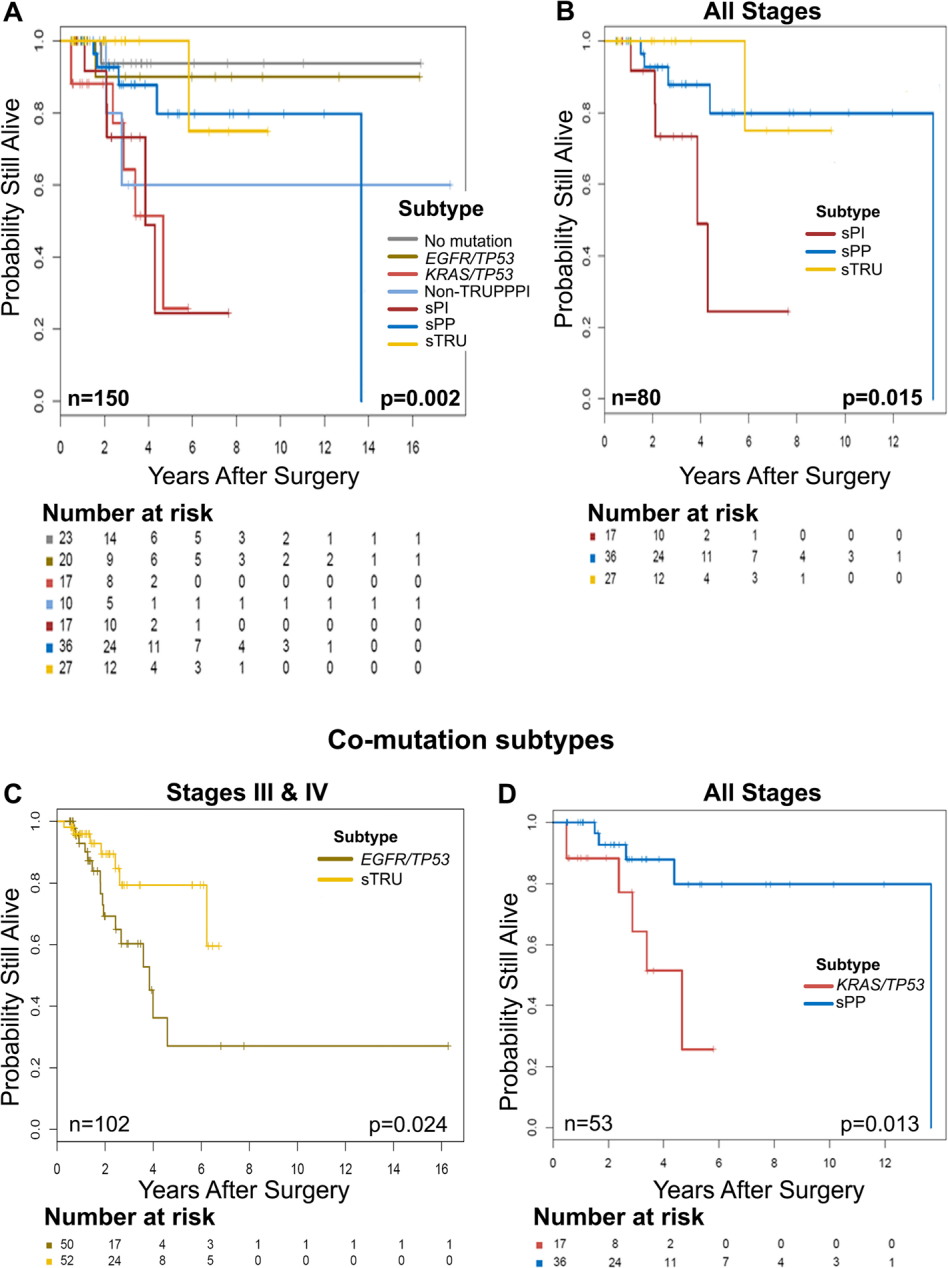
Overall survival (OS) in patients undergoing surgery stratified by simplified molecular subtypes. Kaplan-Meier plots show the prognostic significance of the simplified molecular subtypes in patients who underwent surgery. **a** OS significantly differs between the simplified molecular subtypes. **b** sPI shows worse OS than sTRU and sPP when including all tumor stages. **c** OS significantly differs between the sTRU and *EGFR/TP53* subtypes only in late-stage tumors. **d** OS significantly differs between the sPP and *KRAS/TP53* subtypes in all tumor stages. The number of patients and the log-rank test *p* value are shown at the bottom of each plot

We assessed overall survival in the validation cohort as previously described. The median follow-up time was 1.87 years (interquartile range: 0.9–3.5 years). The median survival time was 5.93 (95% CI: 4.57-not reached) years. We fitted a multivariable Cox proportional hazards regression model to assess for associations between OS and the covariates of age, sex, alcohol intake, stage, molecular subtype, and treatment (surgery, radiation, and/or chemotherapy), selected on the basis of univariate analyses with a cutoff *p* value of 0.25. We observed that patients with older age (hazard ratio [HR] = 1.03, 95% CI: 1.01–1.05) and stage IV disease (HR = 6.51, 95% CI: 2.49–17.04, *p* = 0.006) had worse OS. Although the difference was not statistically significant, patients in the sTRU subtype had better OS (HR = 0.42, 95% CI: 0.18–1.00, *p* = 0.051) whereas patients in the *KRAS*/*TP53* subtype had worse OS (HR = 2.15, 95% CI: 1.02–4.53, *p* = 0.043) than those in the other subtypes (Fig. 2a and b). Patients who underwent surgical resection (HR = 0.33, 95%CI: 0.18–0.60, *p* < 0.001) had better OS than those who did not have surgery. Figure 3a shows significant differences in OS within this subset of patients. We observed statistically significant differences in OS between the sTRU, sPP, and sPI subtypes (Fig. 3b), however, differences between these subtypes when categorized in early stages I and II or late stages III and IV did not reach statistical significance (not shown). Interestingly, no significant differences in OS were observed in patients who received chemotherapy, and OS was significantly worse in those who received radiation therapy, regardless of molecular subtype (HR = 1.87, 95% CI: 1.18–2.96, *p* = 0.007). Notably, OS did not significantly differ between the sTRU and *EGFR/TP53* subtypes in all patients (*p* = 0.84; not shown) or in the patients with early stage tumors (i.e. stages I and II; *p* = 0.16; not shown) who underwent surgery. Statistically significant OS difference between these two subtypes was only observed in late-stage tumors (i.e. stages III and IV; *p* = 0.024; Fig. 3c). Conversely, compared to the sPP subtype, the *KRAS/TP53* subtype showed worse OS both among all patients (*p* = 0.032; Fig. 2b) and among those who underwent surgery (*p* = 0.013; Fig. 3d) regardless of stage.

## Discussion

In this study, we show that the mutational status of three commonly mutated genes can be utilized to create a simplified, mutually exclusive molecular subtype classification of lung adenocarcinomas based on molecular subtypes previously identified using gene expression profiling or larger gene mutation panels and that this simplified classification shows a relationship with prognosis**,** especially in patients who have undergone surgery.

The simplified classification showed high concordance with most previously reported associations, but there were some notable differences. For example, most patients in the sTRU subtype had advanced-stage disease. Among advanced-stage cases, however, those with sTRU subtype had a better prognosis, perhaps a reflection of our referral patient population, whose disease often has not responded to first-line therapy and who present with high-grade, advanced-stage tumors. As expected, the sTRU subtype was also enriched for Asian patients with better prognosis and lepidic histology [8, 12, 22]. Our results also suggest that adenocarcinoma histologic types do not correlate with stage, in keeping with previous findings [12]. However, we observed significant associations between some of the simplified molecular subtypes and morphology. As suggested by Nakaoku et al. and others [23, 24], the PP subtype as well as our sPP subtype are associated with mucinous histology. While the sTRU subtype was not associated with lepidic histology as the TRU is [25], the sTRU however, was associated with non-mucinous tumors. *KRAS/TP53*-mutated tumors more often had solid histology; similarly, a study by Rekhtman et al. [24] found a significant association between a subset of *KRAS*-mutated tumors and solid histology; however, they did not test for *TP53* mutations. Our observations suggest that this association could be unique to *KRAS/TP53* co-mutated tumors.

Interestingly, acinar histology was common in tumors that did not show mutations in this study. Since our NGS panels were developed to target specific exons and did not provide whole-exome/genome results, the no-mutation group could harbor infrequent intronic or exonic mutations/variants in *EGFR*, *KRAS*, *TP53,* and/or other genes. Whereas genomic alterations have been found in all tumors tested by various groups [26, 27], tumors with rare alterations of currently unknown significance could represent unique subtypes where oncogenesis is not driven by common mutations in known genes and genetic pathways [28].

The prognostic difference between the sTRU and *EGFR/TP53* subtypes was only observed in late-stage tumors in our cohort, in keeping with prior findings [29] that have been validated in a recent meta-analysis [30]. Contrariwise, the prognostic difference between the sPP and *KRAS/TP53* subtypes was statistically significant across all groups regardless of tumor stage. We would like to emphasize the distinction of the *KRAS/TP53* molecular subtype because it appears to confer the poorest OS. A report from our group demonstrated different subtypes within *KRAS*-mutated cases and further supports that *KRAS/TP53* co-mutation portends the worst prognosis [31]. Furthermore, recognition of this simplified molecular subtype is supported by recent data showing a potential predictive value of the *KRAS/TP53* subtype for response to PD-1 blockade immunotherapy [32].

Our findings concord and confirm with previous findings that radiation [33] and chemoradiotherapy carry a worse OS with more toxicity and a higher rate of death during treatment, particularly in older patients [34]. Our work thus builds upon recent evidence suggesting radiation therapy be reconsidered in patients with lung adenocarcinoma.

In keeping with the recent recommendations by the updated molecular testing guidelines for the selection of lung cancer patients for targeted therapy, our results provide additional support for the use of cytology specimens as a valuable sample source for molecular testing in patients with lung adenocarcinoma. NGS further enables testing of FNA material and helps avoid the potential risks associated with surgical biopsies [35–42].

Others have shown that gene expression profiling using microarray technology reliably estimates prognosis [9, 16], but the use of microarrays in the clinical setting is limited by the large number of analyzed genes, complex methods, independent validation of the results, low inter-laboratory reproducibility, high cost, long turnaround time, and the need for fresh or frozen tissue [43]. By creating mutually exclusive groups based on easily accessible data, such as *EGFR*, *KRAS*, and *TP53* status, the classification of lung adenocarcinomas into prognostic molecular subtypes could become readily available in routine clinical practice. While oversimplification is a potential limitation of the classification proposed, we believe this simplified classification provides useful prognostic information while retaining the updated proposed nomenclature (i.e., TRU, PP, and PI). This simplified approach will make it easier for molecular genetics laboratories and clinicians to accurately classify patients and will help maintain consistency across different molecular laboratories employing NGS platforms for genomic analysis. Because of the increasing demand for multigene testing over single-gene tests [42] and because most, available NGS panels testing lung adenocarcinoma samples contain these three key genes, we suggest that this simplified classification be used primarily for results obtained via NGS.

## Conclusion

In summary, using mutational data for *EGFR*, *KRAS*, and *TP53*, we have defined prognostic groups similar to those previously identified by more complex genomic methods in patients with lung adenocarcinomas.

## Supplementary information

**Additional file 1.** Supplement Data. Materials and Methods.

**Additional file 2.** Supplement Data. Mutations in Validation Cohort.

## Data Availability

All data generated or analyzed during this study are available upon request.

## Abbreviations

FISH: Fluorescence in situ hybridization
HR: Hazard ratio
NGS: Next-generation sequencing
OS: Overall survival
PCR: Polymerase chain reaction
sPI: Simplified proximal-inflammatory
sPP: Simplified proximal-proliferative
sTRU: Simplified terminal respiratory unit

## References

[1] Lamb D (1984). Histological classification of lung cancer. Thorax..

[2] Siegel RL, Miller KD, Jemal A (2017). Cancer statistics, 2017. CA Cancer J Clin.

[3] Witschi H (2001). A short history of lung cancer. Toxicol Sci.

[4] Dela Cruz CS, Tanoue LT, Matthay RA (2011). Lung cancer: epidemiology, etiology, and prevention. Clin Chest Med.

[5] Herbst RS, Heymach JV, Lippman SM (2008). Lung cancer. N Engl J Med.

[6] Pinsky PF, Church TR, Izmirlian G, Kramer BS (2013). The National Lung Screening Trial: results stratified by demographics, smoking history, and lung cancer histology. Cancer..

[7] Bhattacharjee A, Richards WG, Staunton J, Li C, Monti S, Vasa P (2001). Classification of human lung carcinomas by mRNA expression profiling reveals distinct adenocarcinoma subclasses. Proc Natl Acad Sci U S A.

[8] Cancer Genome Atlas Research N (2014). Comprehensive molecular profiling of lung adenocarcinoma. Nature..

[9] Endoh H, Tomida S, Yatabe Y, Konishi H, Osada H, Tajima K (2004). Prognostic model of pulmonary adenocarcinoma by expression profiling of eight genes as determined by quantitative real-time reverse transcriptase polymerase chain reaction. J Clin Oncol.

[10] Faruki H, Mayhew GM, Serody JS, Hayes DN, Perou CM, Lai-Goldman M (2017). Lung adenocarcinoma and squamous cell carcinoma gene expression subtypes demonstrate significant differences in tumor immune landscape. J Thorac Oncol.

[11] Gordon GJ, Richards WG, Sugarbaker DJ, Jaklitsch MT, Bueno R (2003). A prognostic test for adenocarcinoma of the lung from gene expression profiling data. Cancer Epidemiol Biomarkers Prev.

[12] Hayes DN, Monti S, Parmigiani G, Gilks CB, Naoki K, Bhattacharjee A (2006). Gene expression profiling reveals reproducible human lung adenocarcinoma subtypes in multiple independent patient cohorts. J Clin Oncol.

[13] Parmigiani G, Garrett-Mayer ES, Anbazhagan R, Gabrielson E (2004). A cross-study comparison of gene expression studies for the molecular classification of lung cancer. Clin Cancer Res.

[14] Yatabe Y (2010). EGFR mutations and the terminal respiratory unit. Cancer Metastasis Rev.

[15] Wilkerson MD, Yin X, Walter V, Zhao N, Cabanski CR, Hayward MC (2012). Differential pathogenesis of lung adenocarcinoma subtypes involving sequence mutations, copy number, chromosomal instability, and methylation. PLoS One.

[16] Chen HY, Yu SL, Chen CH, Chang GC, Chen CY, Yuan A (2007). A five-gene signature and clinical outcome in non-small-cell lung cancer. N Engl J Med.

[17] Hernan MA, Clayton D, Keiding N (2011). The Simpson's paradox unraveled. Int J Epidemiol.

[18] Singh RR, Patel KP, Routbort MJ, Reddy NG, Barkoh BA, Handal B (2013). Clinical validation of a next-generation sequencing screen for mutational hotspots in 46 cancer-related genes. J Mol Diagn.

[19] Singh RR, Patel KP, Routbort MJ, Aldape K, Lu X, Manekia J (2014). Clinical massively parallel next-generation sequencing analysis of 409 cancer-related genes for mutations and copy number variations in solid tumours. Br J Cancer.

[20] Kanagal-Shamanna R, Portier BP, Singh RR, Routbort MJ, Aldape KD, Handal BA (2014). Next-generation sequencing-based multi-gene mutation profiling of solid tumors using fine needle aspiration samples: promises and challenges for routine clinical diagnostics. Mod Pathol.

[21] Team RC (2013). R: a language and environment for statistical computing.

[22] Ringner M, Staaf J (2016). Consensus of gene expression phenotypes and prognostic risk predictors in primary lung adenocarcinoma. Oncotarget..

[23] Nakaoku T, Tsuta K, Ichikawa H, Shiraishi K, Sakamoto H, Enari M (2014). Druggable oncogene fusions in invasive mucinous lung adenocarcinoma. Clin Cancer Res.

[24] Rekhtman N, Ang DC, Riely GJ, Ladanyi M, Moreira AL (2013). KRAS mutations are associated with solid growth pattern and tumor-infiltrating leukocytes in lung adenocarcinoma. Mod Pathol.

[25] West L, Vidwans SJ, Campbell NP, Shrager J, Simon GR, Bueno R (2012). A novel classification of lung cancer into molecular subtypes. PLoS One.

[26] Jamal-Hanjani M, Wilson GA, McGranahan N, Birkbak NJ, Watkins TBK, Veeriah S (2017). Tracking the evolution of non-small-cell lung Cancer. N Engl J Med.

[27] Seo JS, Ju YS, Lee WC, Shin JY, Lee JK, Bleazard T (2012). The transcriptional landscape and mutational profile of lung adenocarcinoma. Genome Res.

[28] Swanton C, Govindan R (2016). Clinical implications of genomic discoveries in lung Cancer. N Engl J Med.

[29] Clinical Lung Cancer Genome P, Network Genomic M (2013). A genomics-based classification of human lung tumors. Sci Transl Med.

[30] Zhang R, Tian P, Chen B, Wang T, Li W (2019). The prognostic impact of TP53 comutation in EGFR mutant lung cancer patients: a systematic review and meta-analysis. Postgrad Med.

[31] Skoulidis F, Byers LA, Diao L, Papadimitrakopoulou VA, Tong P, Izzo J (2015). Co-occurring genomic alterations define major subsets of KRAS-mutant lung adenocarcinoma with distinct biology, immune profiles, and therapeutic vulnerabilities. Cancer Discov.

[32] Dong ZY, Zhong WZ, Zhang XC, Su J, Xie Z, Liu SY (2017). Potential predictive value of TP53 and KRAS mutation status for response to PD-1 blockade immunotherapy in lung adenocarcinoma. Clin Cancer Res.

[33] Pezzi TA, Mohamed AS, Fuller CD, Blanchard P, Pezzi C, Sepesi B (2017). Radiation therapy is independently associated with worse survival after R0-resection for stage I-II non-small cell lung Cancer: an analysis of the National Cancer Data Base. Ann Surg Oncol.

[34] Stinchcombe TE, Zhang Y, Vokes EE, Schiller JH, Bradley JD, Kelly K (2017). Pooled analysis of individual patient data on concurrent Chemoradiotherapy for stage III non-small-cell lung Cancer in elderly patients compared with younger patients who participated in US National Cancer Institute cooperative group studies. J Clin Oncol.

[35] Hagiwara K, Kobayashi K (2013). Importance of the cytological samples for the epidermal growth factor receptor gene mutation test for non-small cell lung cancer. Cancer Sci.

[36] Roy Chowdhuri S, Hanson J, Cheng J, Rodriguez-Canales J, Fetsch P, Balis U (2012). Semiautomated laser capture microdissection of lung adenocarcinoma cytology samples. Acta Cytol.

[37] Roy-Chowdhuri S, Chen H, Singh RR, Krishnamurthy S, Patel KP, Routbort MJ (2017). Concurrent fine needle aspirations and core needle biopsies: a comparative study of substrates for next-generation sequencing in solid organ malignancies. Mod Pathol.

[38] Roy-Chowdhuri S, Chow CW, Kane MK, Yao H, Wistuba II, Krishnamurthy S (2016). Optimizing the DNA yield for molecular analysis from cytologic preparations. Cancer Cytopathol.

[39] Roy-Chowdhuri S, Goswami RS, Chen H, Patel KP, Routbort MJ, Singh RR (2015). Factors affecting the success of next-generation sequencing in cytology specimens. Cancer Cytopathol.

[40] Roy-Chowdhuri S, Roy S, Monaco SE, Routbort MJ, Pantanowitz L (2017). Big data from small samples: informatics of next-generation sequencing in cytopathology. Cancer..

[41] Roy-Chowdhuri S, Stewart J (2016). Preanalytic variables in cytology: lessons learned from next-generation sequencing-the MD Anderson experience. Arch Pathol Lab Med.

[42] Lindeman NI, Cagle PT, Aisner DL, Arcila ME, Beasley MB, Bernicker EH (2018). Updated molecular testing guideline for the selection of lung Cancer patients for treatment with targeted tyrosine kinase inhibitors: guideline from the College of American Pathologists, the International Association for the Study of Lung Cancer, and the Association for Molecular Pathology. Arch Pathol Lab Med.

[43] Ramaswamy S (2004). Translating cancer genomics into clinical oncology. N Engl J Med.

