# Supplementary material for: Simplified molecular classification of lung adenocarcinomas based on EGFR, KRAS, and TP53 mutations
Source: BMC Cancer. 2020 Jan 31;20:83. doi: 10.1186/s12885-020-6579-z (PMC6995064; doi:10.1186/s12885-020-6579-z)
Supplement: Supplementary file 1 — Additional file 1. Supplement Data. Materials and Methods. [file 12885_2020_6579_MOESM1_ESM.docx]

**Simplified Molecular Classification of Lung Adenocarcinomas Based on EGFR, KRAS, and TP53 Mutations**

**Supplement Data**

**Materials and Methods**

*Mutational analysis*

PCR testing for *BRAF* and *KRAS* was performed using pyrosequencing. For *BRAF* exon 15 (codon 595/596 and codon 599/600) mutations, a 231-base pair amplicon was amplified using a forward (M13-CAT AAT GCT TGC TCT GAT AGG A) and a reverse biotinylated primer (biotin-M13-GGC CAA AAA TTT AAT CAG TGG A). For *KRAS* hotspot mutations in codons 12 and 13, a 98-base pair amplicon was amplified using a forward (TAT AAA CTT GTG GTA GTT GG) and a reverse biotinylated primer (biotin-ATT GTT GGA TCA TAT TCG T). For codon 61 a forward (TTG GAT ATT CTC GAC ACA) and reverse biotinylated primer (biotin-CCA CCT ATA ATG GTG AAT ATC T) were used. The PCR master mix for both genes contained the forward and reverse primers (each 10 μmol/L), 10 mmol/L of dNTP mix, 25 mmol/L of magnesium chloride, ×10 PCR buffer (Applied Biosystems, Carlsbad, CA), 5 U/μL of Taq Gold and 200 ng of sample genomic DNA in a total volume of 48 μL. Subsequently, PCR amplification for both target genes was performed on an ABI 2720 Thermocycler (Applied Biosystems, USA) with the following cycling conditions: initial denaturing at 95ºC for 12 minutes, 50 cycles at 94ºC for 30 seconds, 55ºC for 30 seconds, and 72ºC for 30 seconds, and final extension at 72ºC for 10 minutes. Appropriate positive (melanoma cell line A375, for *BRAF* exon 15 and prior positive sample for *KRAS*), negative (control sample), and reagent controls were included. The PCR products underwent electrophoresis on agarose gels to confirm successful amplification of the PCR products before pyrosequencing. PCR products (each 15 μL) were then sequenced in duplicate using the pyrosequencing PSQ96 HS System (Biotage AB, Uppsala, Sweden) per manufacturer’s instructions.

Mutations in exons 18 to 21 of *EGFR* were assessed using Sanger sequencing using the following primers: exon 18 forward (M13-CAA GTG CCC TGT CCT GGC ACC CAA GC) and reverse (M13-CCA AAC ACT CAG TGA AAC AAA GAG), exon 19 forward (M13-GTA ACA TCC ACC CAG ATC) and reverse (M13-GTC TAG AGC AGA GCA GCT G), exon 20 forward (M13-GAA ACT CAA GAT CGC ATT CAT GC) and reverse (M13-GCA AAC TCT TGC TAT CCC AGG AG), and exon 21 forward (M13-CAG CCA TAA GTC CTC GAC GTG G) and reverse (M13-CAT CCT CCC CTG GAT GTG TTA AAC). The PCR master mix was amplified as described above using the following cycling conditions: initial denaturing at 95ºC for 10 minutes, 50 cycles at 94ºC for 1 minute, 60ºC for 1 minute, and 72ºC for 1 minute, and final extension at 72ºC for 10 minutes. Appropriate positive (previously positive patient sample), negative (Tijerina cell line negative for *EGFR* mutation), and reagent controls were included. The PCR products underwent electrophoresis on agarose gels to confirm successful amplification before Sanger sequencing using the ABI PRISM 3130 Genetic Analyzer (Applied Biosystems, USA) per manufacturer’s instructions.
